# Oxygen therapy attenuates neuroinflammation after spinal cord injury

**DOI:** 10.1186/s12974-023-02985-6

**Published:** 2023-12-19

**Authors:** Michael D. Sunshine, Victoria E. Bindi, Branden L. Nguyen, Vivian Doerr, Franccesco P. Boeno, Vijayendran Chandran, Ashley J. Smuder, David D. Fuller

**Affiliations:** 1 Department of Physical Therapy, University of Florida, Gainesville, FL USA; 2 Breathing Research and Therapeutics Center, University of Florida, Gainesville, FL USA; 3 McKnight Brain Institute, University of Florida, Gainesville, FL USA; 4 Department of Pediatrics, University of Florida, Gainesville, FL USA; 5 Department of Applied Physiology and Kinesiology, University of Florida, Gainesville, FL USA

**Keywords:** Hyperbaric oxygen, Hyperoxia, Spinal cord injury

## Abstract

Acute hyperbaric O_2_ (HBO) therapy after spinal cord injury (SCI) can reduce inflammation and increase neuronal survival. To our knowledge, it is unknown if these benefits of HBO require hyperbaric *vs.* normobaric hyperoxia. We used a C4 lateralized contusion SCI in adult male and female rats to test the hypothesis that the combination of hyperbaria and 100% O_2_ (i.e. HBO) more effectively mitigates spinal inflammation and neuronal loss, and enhances respiratory recovery, as compared to normobaric 100% O_2_. Experimental groups included spinal intact, SCI no O_2_ therapy, and SCI + 100% O_2_ delivered at normobaric pressure (1 atmosphere, ATA), or at 2- or 3 ATA. O_2_ treatments lasted 1-h, commenced within 2-h of SCI, and were repeated for 10 days. The spinal inflammatory response was assessed with transcriptomics (RNAseq) and immunohistochemistry. Gene co-expression network analysis showed that the strong inflammatory response to SCI was dramatically diminished by both hyper- and normobaric O_2_ therapy. Similarly, both HBO and normobaric O_2_ treatments reduced the prevalence of immunohistological markers for astrocytes (glial fibrillary acidic protein) and microglia (ionized calcium binding adaptor molecule) in the injured spinal cord. However, HBO treatment also had unique impacts not detected in the normobaric group including upregulation of an anti-inflammatory cytokine (interleukin-4) in the plasma, and larger inspiratory tidal volumes at 10-days (whole body-plethysmography measurements). We conclude that normobaric O_2_ treatment can reduce the spinal inflammatory response after SCI, but pressured O_2_ (i.e., HBO) provides further benefit.

**Supplementary Information:**

The online version contains supplementary material available at 10.1186/s12974-023-02985-6.

## Introduction

Local tissue hypoxia is one factor contributing to tissue damage and impaired recovery after spinal cord injury (SCI) [1, 2]. The tissue partial pressure of oxygen (PO_2_) at or near an acute spinal cord lesion rapidly drops close to zero [1]. Even after chronic SCI, impaired blood flow and chronically low spinal PO_2_ remain [2]. Treating low tissue PO_2_ can be achieved by raising the PO_2_ of inspired air. This increases alveolar, and therefore plasma PO_2_, creating a gradient that favors O_2_ diffusion from the vasculature into the injured spinal cord. Breathing 100% O_2_ at normobaric pressure (*i.e.*, one atmosphere or ATA) can increase spinal PO_2_ after SCI [2], and PO_2_ can be further increased by providing pressurized 100% O_2_ (i.e., hyperbaric O_2_ therapy, or HBO). For example, breathing 100% O_2_ at three atmospheres (ATA) pressure can raise plasma PO_2_ from ~ 100 to > 2000 mmHg [3], and in turn this can drive spinal PO_2_ to ~ 500 mmHg to potentially mitigate local tissue hypoxia in the injured spinal cord [1].

Prior studies have demonstrated benefit of HBO therapy after SCI. Experiments using rodent SCI models show that HBO can reduce inflammation [4] and apoptosis [5], preserve neurons [6] and promote muscle health [7]. Further, multiple studies have treated spinally injured humans with HBO, initiated as early as 24 h post-injury [8–13]. Some degree of neurological benefit has been reported in most of the published clinical trials, although rigorous control groups are lacking. Collectively, the aforementioned data show that HBO therapy conveys benefit in SCI animal models, and may be a clinically viable treatment after SCI. However, HBO therapy consists of two primary variables: elevated ambient pressure (i.e., hyperbaria) and elevated O_2_ (i.e., hyperoxia). One of the first studies of O_2_ therapy after SCI reported that acutely (minutes) after SCI, hyperoxia alone (i.e., normobaric 100% O_2_) was not sufficient to affect the PO_2_ of the injured spinal cord [1]. However, a more recent study demonstrates that inspiring 100% O_2_ at ambient pressure can attenuate spinal pathology after chronic SCI [2]. To our knowledge, no study to date has verified that both hyperbaria and hyperoxia are necessary to convey HBO therapy benefits in the days-weeks following SCI. This is a fundamentally important question for optimization of O_2_ treatment for SCI, particularly since normobaric O_2_ therapy is (relatively) easily administered, even in an acute care setting, whereas acute hyperbaric therapy presents considerable logistical hurdles.

We used a C4 lateralized contusion SCI in adult male and female rats to test the hypothesis that the combination of hyperbaria and 100% O_2_ (i.e. HBO) more effectively mitigates spinal inflammation and neuronal loss, and enhances respiratory recovery, compared to normobaric 100% O_2_. A pressure control group was also included to provide normoxic blood gas conditions but at increased atmospheric pressure. Breathing is virtually always impaired after cervical SCI, and respiratory-related deficits are the leading contributor to morbidity and mortality [14]. Thus, breathing was evaluated as part of a comprehensive battery of outcome measures that included transcriptomics (RNA-seq) of the injured spinal cord, spinal cord histology, serum cytokines, inspiratory tidal volume and respiratory rate, and diaphragm muscle function.

## Methods

### Overview

**Fig. 1:**
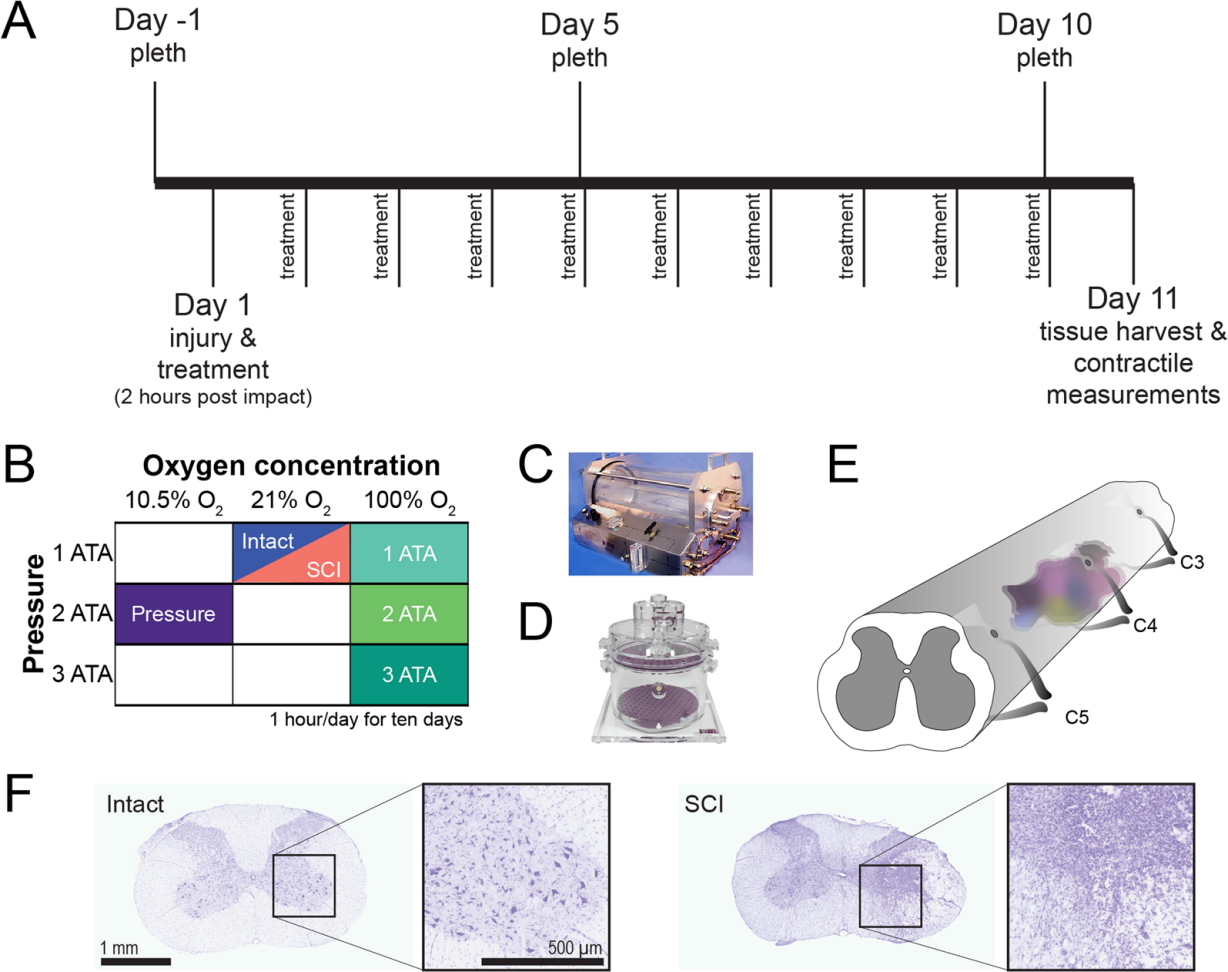
Overview of the study design. **A** Study timeline indicating daily O_2_ treatments and plethysmography (pleth) measurements. **B** Schematic indicating the treatments received by each of the experimental groups. Each of the colors indicates a different experimental group. **C** The 32 L hyperbaric chamber used for treating rats with pressurized gas. **D** The whole body plethysmography chamber used for recording breathing. **E** Drawing of the spinal cord illustrating the approximate location and size of the lesion induced by unilateral contusion at C4. **F** Representative photomicrographs of transverse (cross) sections of the C4 spinal cord stained with cresyl violet. Examples are included from an intact (uninjured) cord (left) and after C4 contusion (SCI, right)

Approval to study adult male and female Sprague–Dawley rats was provided by the University of Florida animal care and use committee and conformed to NIH guidelines. Rats had access to food and water ad libitum throughout the study. The overall experimental design is presented in Fig. 1A, B. Rats were receive O_2_ treatments in a 32 L chamber (Fig. 1C). Breathing was assessed in awake rats using whole body plethysmography (Fig. 1D). One experimental group had laminectomy surgery (spinal intact, *n* = 18, 9 male). Five experimental groups had cervical SCI (Fig. 1E, F). One SCI group (*n* = 19, 10 male) and the spinal intact group were exposed to normobaric (1 ATA) normoxic gas (21% O_2_). A SCI pressure control group was treated with 10.5% O_2_ at 2 ATA (*n* = 17, 10 male), to produce an approximately normoxic blood O_2_ level, but at increased atmospheric pressure [15]. The O_2_ therapy groups were exposed to 100% O_2_ at 1 (*n* = 18, 9 male), 2 (*n* = 19, 10 male), or 3 ATA (*n* = 21, 9 male).

### Spinal cord injuries

Rats were anesthetized with ketamine (80 mg/kg) and xylazine (10 mg/kg; i.p.). Injuries were performed in the same manner as previous reports [16, 17]. The skin was incised above the cervical spinal cord to expose the musculature over C2-C6. The muscles were reflected back and the vertebrae were cleaned with a curette to enable clamping. A laminectomy was performed on the right side of the fourth cervical (C4) vertebra and the animals we held in stabilization clamps at C3 and C6. We then used the Infinite Horizons impactor with a 2.5 mm tip to deliver a 150 kdyn injury on the right side of the spinal cord. A total of 112 animals were included in the study. Cohort 1 focused on breathing measurements and histological evaluation of the spinal cord and had *n* = 81. Cohort 2 was used for spinal RNAseq measurements, and had *n* = 21.

### O_2_ exposures

All rats were placed in a 32 L chamber (Fig. 1C) for one hour per day for 10 consecutive days. The chamber was first flushed with 100% O_2_ at six liters per minute (LPM), followed by sustained flow at 3 LPM. Animals began treatment approximately two hours after SCI (average of 126 ± 31 min).

### Plethysmography

To assess breathing recovery following SCI we utilized whole body plethysmography. Animals were acclimated to the chambers on the day prior to taking the pre-injury measurement. A pre-injury measurement was recorded 2–3 days before SCI surgery and on day 5- and 10-post injury. Plethysmography recordings on treatment days were performed prior to administering the treatment. Plethysmography sessions consisted of an hour of normoxic baseline breathing (21% O_2_ / 79% N_2_), followed by a ten-minute hypoxic-hypercapnic (10% O_2_, 7% CO_2_) challenge to test the capacity to increase breathing [18–20]. The last five minutes of data within the normoxic and hypoxic-hypercapnic periods was used for calculating tidal volume, breathing rate, and minute ventilation. In addition to these traditional ventilation metrics we performed a respiratory waveform cluster analysis [18]. As this analysis is intended to investigate subtle differences in respiratory waveform patterns, we analyzed the entire one-hour normoxic period.

### Tissue harvesting

Animals in cohort 1 were injected with 1 mL Beuthanasia-Special-D (phenytoin/pentobarbital) solution. Adequate surgical anesthesia was confirmed by the absence of palpebral and toe-pinch reflexes, and the heart was then was exposed via thoracotomy. Three mL of blood was collected via left ventricle puncture and placed in tripotassium (K3) ethylenediamine tetraacetic acid (EDTA) blood collection tubes (see: plasma cytokine analysis). The diaphragm was removed for in vitro contractility studies and histological assessment [7]. Rats were then transcardially perfused with saline and 4% PFA, and the spinal cord was extracted. Rats in cohort 2 were anesthetized with isoflurane and transcardially perfused with ice cold saline and the spinal cord was harvested and placed in Trizol. Spinal tissue was then processed using an RNAeasy kits (Qiagen # 74004) to isolate RNA [21].

### Spinal cord immunohistochemistry

Following overnight post-fixation in 4% PFA, spinal cords were transferred to 30% sucrose. Once it had sank in the solution, the spinal cord was blocked in optimal cutting temperature (OCT) media and stored at -80 C. Spinal cords were sectioned at 20 µm and cut into a seven-section series to enable staining for multiple markers in serial. Sections were then processed with (1) cresyl violet, or (2) FluoroMyelin, or with primary antibodies against (3) IBA1 (1:300; Wako # 019-19741) and GFAP (1:500; Thermo # 53-9892-82), or (4) NeuN (1:500; Encor #MCA-1B7). Cresyl violet was performed in manner consistent with previous reports [16] and FluoroMyelin was performed per the manufacturer instructions. For immunohistochemistry, sections were processed with heat induced epitope retrieval, permeabilized (0.4% triton; 15 min) and blocked (3% serum, 0.2% triton; 60 min). Following blocking, primary antibodies were incubated overnight at 4°. After primary incubation and three serial washes with 1 × PBS, secondary antibodies were incubated for two hours at room temperature. Secondary antibodies were washed off with 1 × PBS and coverslips were mounted with VECTASHIELD Vibrance (Vector Laboratories).

### Plasma cytokine analysis

Blood samples were centrifuged at 5000 rpm for 10 min at 4 °C. Plasma was separated and immediately stored at − 80 °C until analyzed. Plasma cytokine and chemokine concentrations were measured using a Luminex Magpix® multiplex analyzer (25 Plex MILLIPLEX MAP Rat Cytokine/Chemokine Magnetic Bead Panel, Millipore, Burlington, MA). Samples were run in duplicate, and the assay was analyzed using Belysa™ Immunoassay Curve Fitting Software. Manufacturer instructions were followed for sample preparation.

### Diaphragm contractility

Our methods for measuring ex vivo diaphragm contractility have been described [22, 23]. A strip the midcostal region of the hemi-diaphragm ipsilateral to the SCI was dissected including the tendinous attachments at the central tendon and rib. The strip was suspended vertically between two platinum electrodes within a jacketed tissue bath containing Krebs–Henseleit buffer (Sigma-Aldrich, St. Louis, MO, USA) equilibrated with 95% O_2_–5% CO_2_ at room temperature. Each end was secured, and the central tendon was clamped and attached to a Dual-Mode Muscle Lever System (305-CLR, Aurora Scientific Inc., Aurora, Canada) The muscle strip was stimulated along its length via a biphasic high-power stimulator (701C, Aurora Scientific Inc., Aurora, Canada). Optimal length (L_0_) was determined by increasing the muscle length and measuring tetanic force at 1 Hz stimulation (twitch, 600 mA current, 0.2 ms pulse) until max active force (peak force – baseline force) was achieved (L_0_). At L_0_, the diaphragm strip was stimulated every 2 min at frequencies of 1, 15, 30, 60, 100, and 160 Hz to complete the force-frequency curve. Force was normalized to cross-sectional area (CSA, N/cm^2^) as previously described [22, 23].

### Diaphragm cross sectional analysis

The mid-costal diaphragm was cut in 10 μm sections using a cryostat (HM 550 Cryostat, Thermo Fisher Scientific, Waltham, MA, USA) and stained for laminin (L9393) (Millipore Sigma), myosin heavy chain Type I (A4.840) (Developmental Studies Hybridoma Bank (DSHB), Iowa City, IA, USA) and Myosin Heavy Chain Type IIa (SC-71) (DSHB). Cross sectional area of myofibers was analyzed with Image J software (NIH, Bethesda, MD, USA).

### Spinal RNAseq data analysis

A weighted signed gene co-expression network analysis (WGCNA) was constructed. The aim of the WGCNA was to identify functionally related groups of genes or “modules”. Initially, we computed the Pearson correlation between each pair of selected genes, producing a similarity (correlation) matrix. The adjacency matrix was then calculated by raising the absolute values of the correlation matrix to a power (*β*), as previously described [24]. We chose the parameter *β* using the scale-free topology criterion [24], ensuring the resulting network connectivity distribution best approximated scale-free topology. Next, we used the adjacency matrix to define a measure of node dissimilarity, based on the topological overlap matrix—a biologically meaningful measure of node similarity [24]. We then hierarchically clustered the genes using the distance measure, and determined the modules by choosing a height cutoff for the resulting dendrogram, employing a dynamic tree-cutting algorithm [24]. Through this network analysis, we identified modules of differentially expressed genes across various datasets post-spinal injury and hyperbaric O_2_ therapy, and calculated the first principal component of gene expression in each module (the module eigengene). Subsequently, we correlated the module eigengenes with treatment to select modules for functional validation. The WGCNA package is available online [25], and all analyses were performed in accordance with user manual tutorials. The code for WGCNA analyses can be found online [26]. Gene ontology and pathway enrichment analysis was carried out using the DAVID platform [27] (DAVID, https://david.ncifcrf.gov/). RNAseq datasets are available at https://odc-sci.org/.

### Data analysis and statistics

Data analysis and statistical testing was performed with MATLAB 2020a. Statistical tests are described in the figure legends. For plethysmography data, traditional metrics of ventilation (tidal volume, respiratory rate, and minute ventilation) were calculated from calibrated flow waveforms during the baseline (normoxia) and challenge (hypoxic-hypercapnia) periods [20]. We further analyzed the plethysmography data using our previously published method to evaluate the prevalence of distinct respiratory waveforms [18]. Histological sections were analyzed with custom MATLAB scripts available at https://odc-sci.org/. Photomicrographs were taken using a Keyence BX-10 (10 × objective). Images were first flat fielded to remove illumination artifacts. To flat-field the images we generated a morphological opened image using the imopen command in MATLAB. This opened image was subtracted from the original image. This process effectively high-pass filters the images to remove illumination artifacts from bubbles or stitching artifacts. A blinded user oriented each section so that the dorsal root entry zones were parallel across the section and identified the location of the central canal. The location of the central canal was used to assess regional distribution of cell types. IBA-1 + and GFAP + cells were identified as regions with intensity above a threshold. The threshold was calculated by computing the average intensity of a region of interest on the contralesional side of the section. The average intensity of staining was calculated for all pixels above this cutoff threshold.

NeuN + cell counts were calculated in a similar manner, but we first used freely available “trainable” software to identify autofluorescence regions due to overt tissue damage related to the lesion (https://www.mathworks.com/help/vision/ug/semantic-segmentation-using-deep-learning.html). These regions were excluded from analysis to mitigate false positive counts. Each section was divided into quadrants: ipsilateral-ventral, ipsilateral-dorsal, contralateral-ventral, contralateral-dorsal. We then calculated the size of the detected neurons to stratify them into putative interneurons and putative motor neurons. To ensure appropriate detection of neurons by the code, cell identification masks were assessed in a random manner (approximately every 20th section) by a blinded reviewer.

## Results

### RNAseq analysis of the injured spinal cord

**Fig. 2:**
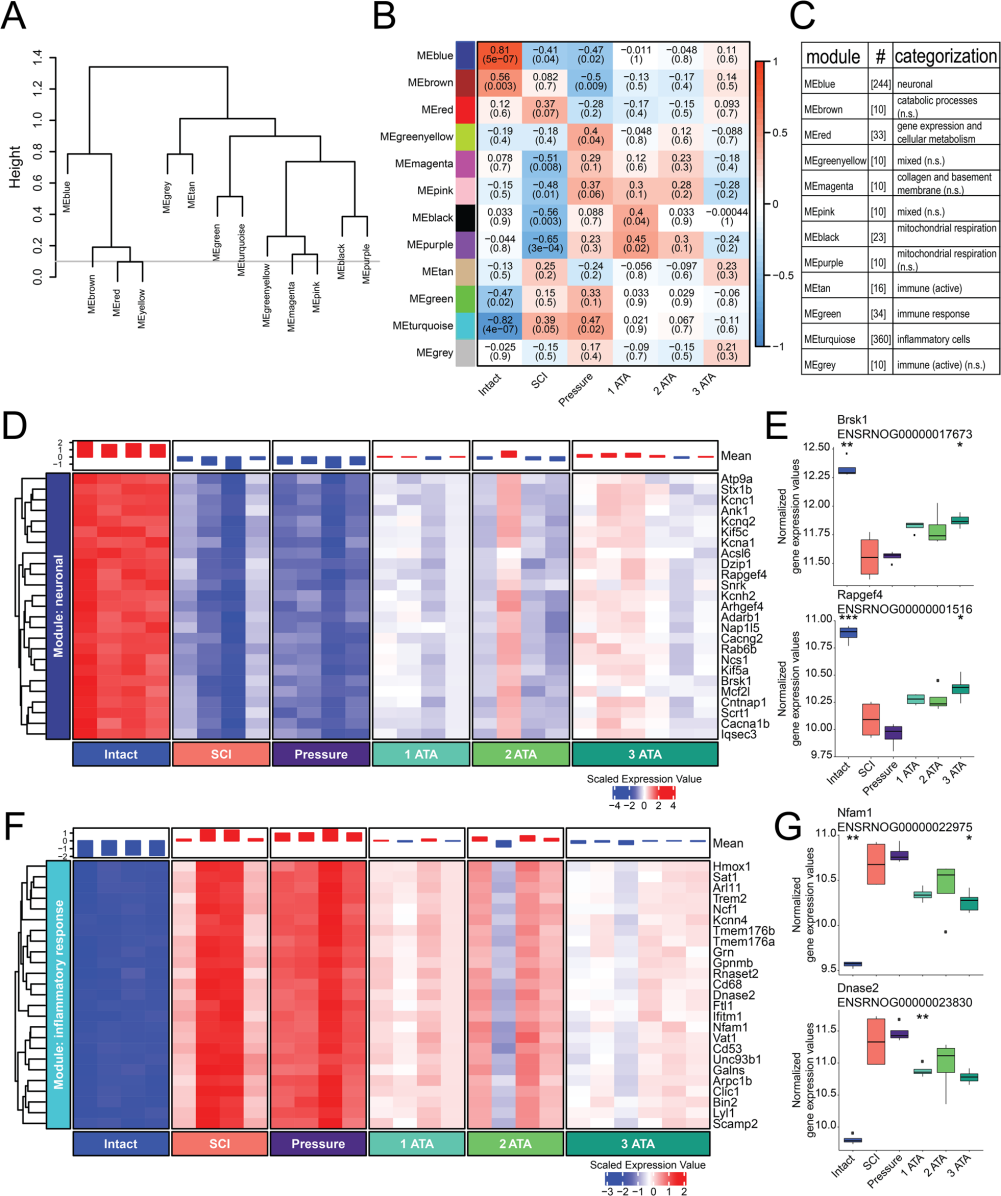
Impact of SCI and O_2_ therapy on the spinal cord transcriptome. **A** Data were generated by performing RNAseq on C4 spinal tissues, followed by hierarchical clustering of Weighted Gene Co-Expression Network Analysis (WGCNA) module eigengenes. In panel **A**, “Height” indicates the relative difference between modules, and the branches of the dendrogram group together eigengenes that are positively correlated. **B** The relative expression of each module, in each experimental group. Each row corresponds to a module eigengene, and each cell contains the corresponding correlation and *p*-value. Red and blue color denote positive and negative correlation with gene expression, respectively. **C** The functional classification of each module and number of associated genes. **D**, **F** Heat maps depict expression of the top 25 hub genes (rows) across animals (columns) for the six experimental groups (red corresponds to gene upregulation and blue to downregulation). These plots are provided for the blue module (“neuronal”, **D**) and turquoise (“inflammatory”, **F**) module. Bar plots above the heat maps show the overall expression level within each animal. **E**, **G** Boxplots are provided to illustrate variability in the expression levels of representative hub genes in the blue (top) and turquoise (bottom) modules. * indicates *p* < 0.05, student’s *t*-test compared to SCI

The impact of SCI and O_2_ therapy on the transcriptome of the injured spinal cord was examined by performing RNAseq on C4 spinal tissue samples. We utilized the Weighted Gene Co-Expression Network Analysis (WGCNA) to identify "gene modules" related to O_2_ therapy following SCI (Fig. 2A–C). WGCNA enables the detection of modules of highly co-expressed genes, reflecting shared biological functions, crucial pathways, and key hub genes within the modules for further validation [28, 29]. The identified gene modules were assigned a color as shown on the left side of Fig. 2B, which also provides a heat map that contrasts gene module expression across the six experimental groups. The functional category of each module is shown in Fig. 2C. Expression of the “neuronal”-blue and “inflammatory response”-turquoise modules were particularly impacted by SCI (Fig. 2D–G). The pressure control treatment did not correct for the change in expression pattern due to SCI within these two gene modules, but all of the O_2_ therapies (1-, 2- and 3ATA) considerably affected the gene expression. For example, the striking downregulation of the neuronal module that occurred after SCI was attenuated in all three of the O_2_ therapy groups (Fig. 2D). Two of the top hub genes from the neuronal module were Brsk1 and Rapgef4 (Fig. 2E), which are associated with neurotransmitter secretion and regulation of action potentials [30, 31]. The inflammatory module showed predictably robust expression after SCI (Fig. 2F). However, rats treated with O_2_ therapy had a distinct attenuation of inflammatory gene expression as shown in Fig. 2F. Two of the top hub genes from the inflammatory module were Nfam1 and Dnase2 (Fig. 2G), both of which are associated with immune responses [32, 33].The impact of SCI and O_2_ therapy on the expression of the remaining ten gene modules that were identified using the gene co-expression network analyses is shown in Additional file 1: Fig. S1.

### Blood serum

A luminex immunoassay was used to evaluate the impact of SCI and O_2_ treatment on blood serum levels of 16 different cytokines at 10 days post-injury. Most noteworthy, interleukin 4 (IL-4) was different across treatment groups (Kruskal–Wallis, *p* = 0.004), with the 3 ATA O_2_ therapy group showing the highest levels. Interleukin 17A was also different across the treatment groups (Kruskal–Wallis, *p* = 0.033), but changes were unique to the normobaric (1 ATA) O_2_ therapy group. Serum levels of leptin were dramatically reduced following SCI in all rats (one-way ANOVA, *p* = 0.0006), with no impact of O_2_ treatment. The results of the entire cytokine panel are provided in Additional file 1: Fig. S2.

### Spinal histological evaluation

**Fig. 3:**
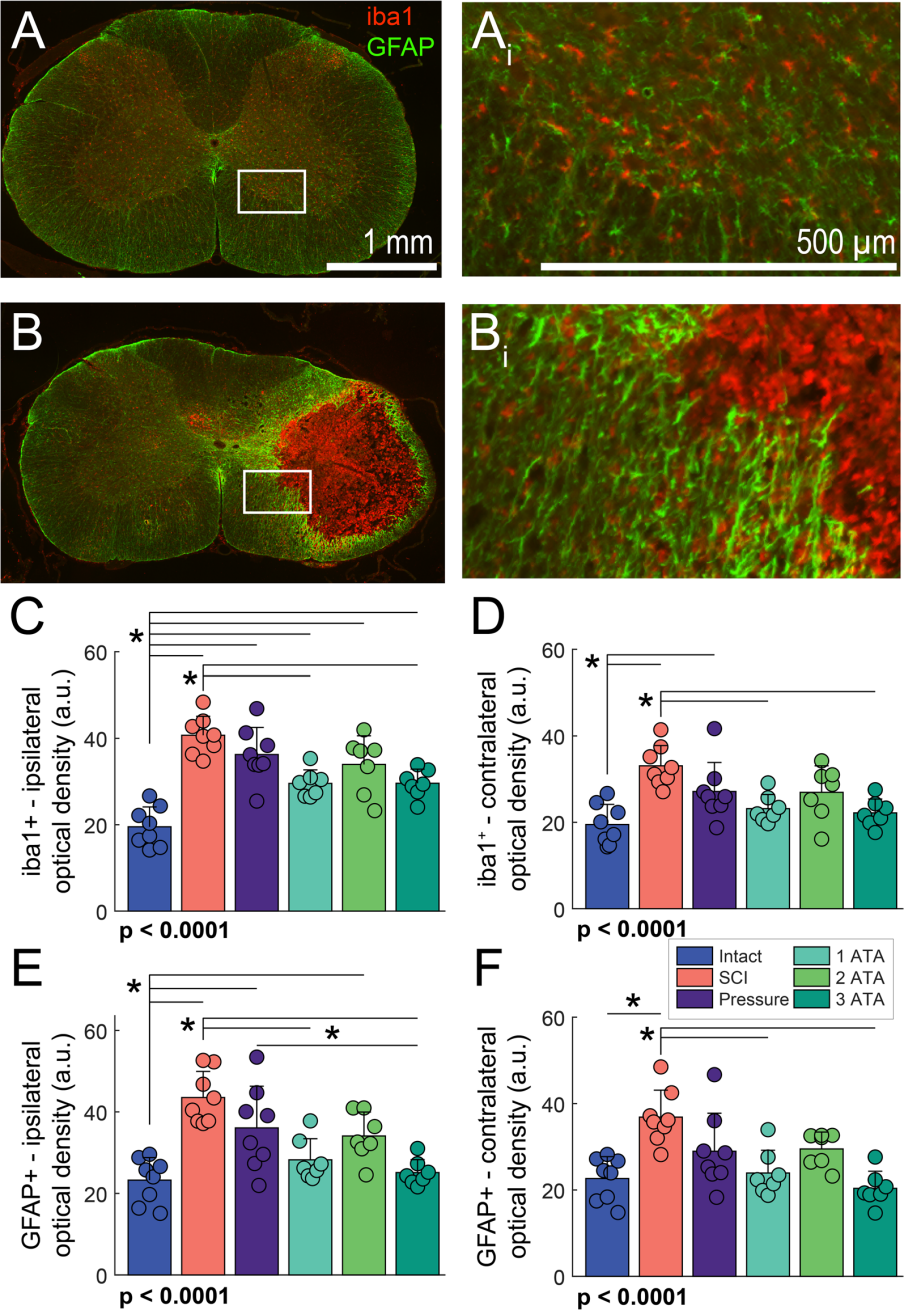
Impact of O_2_ therapy on histological assessment of neuroinflammation near the spinal cord lesion. Representative photomicrographs depicting IBA1 and GFAP immunostaining are shown in C4 spinal cord transverse sections obtained from a spinal intact (panel **A**) and injured rat (SCI group, panel **B**). **Ai** and **Bi** show increased magnification views of the area highlighted by the box in A and B. Panels **C** and **D** provide quantitative evaluation of IBA1 + optical density on the ipsilateral and contralateral sides of the spinal cord, respectively. Immunostaining was evaluated ± 10 mm from C4 (lesion epicenter). Panels **E** and **F** present GFAP + optical density in the ipsilateral and contralateral spinal cord. A one-way analysis of variance was used to compare groups. The omnibus effect of group *p*-value is noted in the figure; *indicates *p* < 0.05 Tukey–Kramer post-hoc compared to Intact

Photomicrographs of cresyl violet stained spinal cord illustrating the lesion are shown in Fig. 1F and Additional file 1: Fig. S3. Antibodies against IBA1 and GFAP were used to identify monocytes/microglia and astrocytes, respectively, in tissue sections from the mid-cervical spinal cord. In the spinal intact condition, IBA1-positive and GFAP-positive cells were distributed throughout mid-cervical white and gray matter (Fig. 3A). Following SCI, intense GFAP staining was present at the edge of the lesion, and IBA1 staining was concentrated within the injury core (Fig. 3B). Quantification of immunostaining optical density was completed for regions ipsilateral and contralateral to the spinal lesion (Fig. 3C–F and Additional file 1: Fig. S4). In the ipsilateral cord, IBA1 optical density was elevated in all groups as compared to spinal intact (Fig. 3C, Tukey–Kramer post-hoc *p* < 0.05). However, SCI groups which received any form of O_2_ therapy tended to have reduced ipsilateral IBA1 optical density as compared to untreated. This reduction was statistically significant in the 1- and 3 ATA O_2_ treatment groups (Fig. 3C, Tukey–Kramer post-hoc *p* < 0.05). Similar trends in IBA1 optical density were present in the spinal cord contralateral to the lesion (Fig. 3D). Thus, IBA1 optical density was greater in the SCI and SCI + pressure groups *vs.* spinal intact (Fig. 3D, Tukey–Kramer post-hoc *p* < 0.05), and O_2_ treatment reduced IBA1 optical density as compared to untreated SCI.

The optical density of GFAP staining ipsilateral to the lesion was markedly elevated in the untreated SCI group compared to the spinal intact condition (Fig. 3E, Tukey–Kramer, *p* < 0.05). Similar to the IBA1 data, the 1- and 3 ATA O_2_ treatment groups showed a statistically significant reduction in GFAP optical density in the ipsilateral spinal cord (Tukey–Kramer, *p* < 0.05, Fig. 3E). The contralateral spinal cord also showed a marked increase in GFAP optical density after SCI. However, O_2_ treatment at 1- and 3 ATA produced a reduction in GFAP staining in the contralateral cord (Fig. 3F, Tukey–Kramer post-hoc *p* < 0.05).

### Tissue vacuolization and neuronal counts

**Fig. 4:**
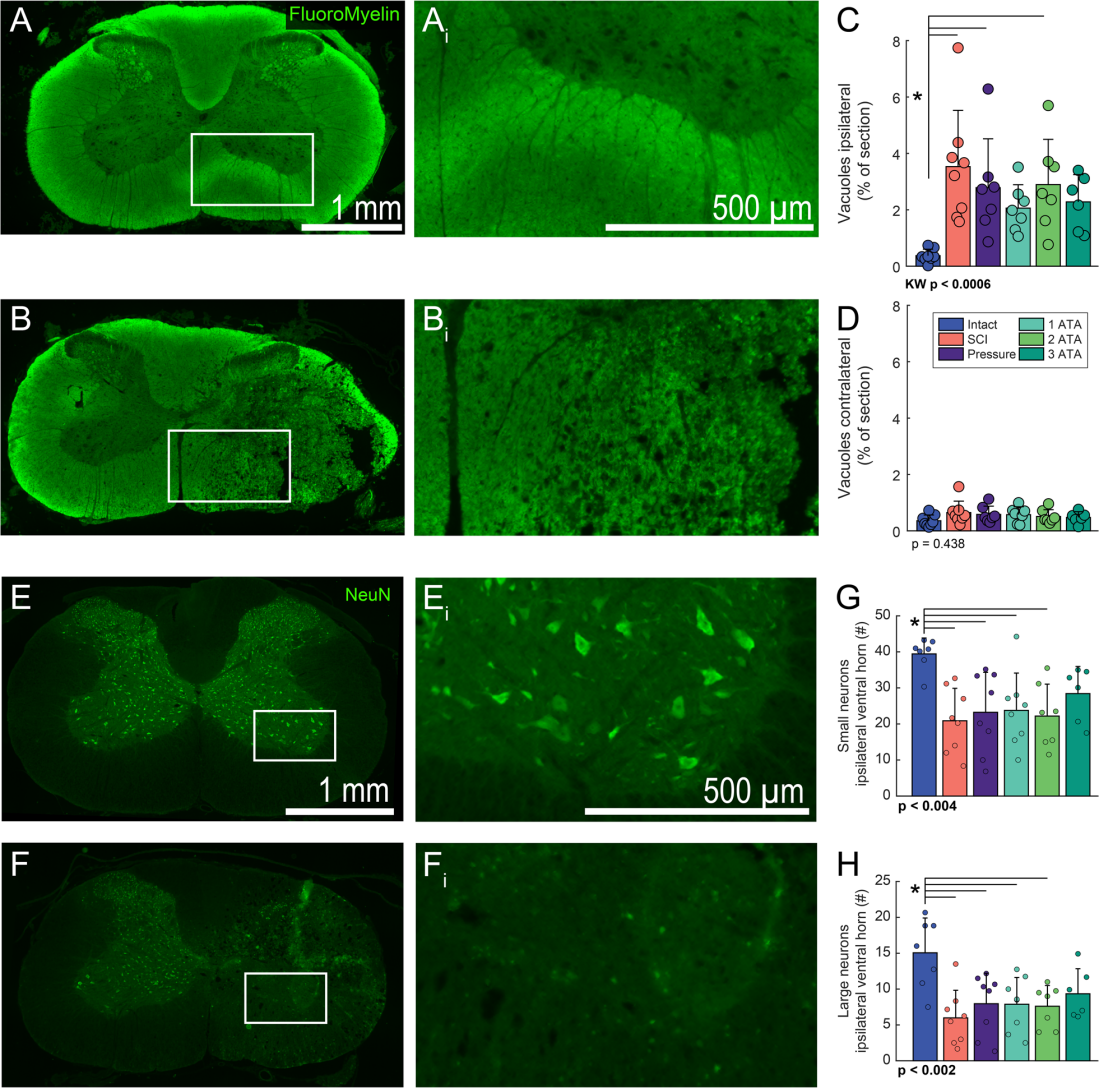
Impact of O_2_ therapy on vacuolization and neuronal numbers in the injured spinal cord. Representative photomicrographs of FluoroMyelin stained transverse C4 spinal cord sections are presented in panel **A** (spinal intact) and panel **B** (SCI group). Panels **Ai** and **Bi** show increased magnification views of the areas highlighted by the box in A and B. Panels B and Bi illustrate the increase in vacuolization in tissues from the injured spinal cord. Mean data were assessed ± 2 mm from the C4 lesion epicenter and are presented for the ipsilateral (panel **C**) and contralateral spinal cord (panel **D**). Panels **E–F** show example photomicrographs of the C4 spinal cord depicting NeuN staining (to recognize neurons) in the spinal intact (E) and injured spinal cord (F). **Ei** and **Fi** show increased magnification views of the areas highlighted by the box in E and F. Panel **G** shows group data for small neurons (23–115 µm^2^) and panel **H** shows large neurons (116–345 µm^2^) in the ventral horn on the ipsilateral side. In both cases, although values are lower, neuronal counts in the 3 ATA group are not statistically different than spinal intact. One-way analysis of variance was used to compare the groups; if a data-set failed ANOVA assumptions a Kruskal–Wallis (KW) test was used. The omnibus effect of group *p*-value is noted in the figure; *indicates *p* < 0.05 Tukey–Kramer post-hoc compared to Intact

Vacuolization of spinal cord tissues, as shown in Fig. 4A, B, was assessed as a marker of white matter health [34] and neuroprotection [35]. After SCI, a dramatic increase in vacuolization was present ipsilateral (Fig. 4C, Additional file 1: Fig. S5, Kruskal–Wallis, *p* < 0.0009) but not contralateral to the lesion (Fig. 4D,   *p* = 0.438). Ipsilaterally, there was an indication of an impact of O_2_ therapy at 1- and 3-ATA. Specifically, both groups showed a decrease in detectable vacuoles when compared to SCI without treatment, and these were not statistically different compared to the spinal intact condition (Fig. 4C).

The NeuN staining procedure effectively labeled spinal neurons as illustrated by the photomicrographs in Fig. 4E, F. Neuronal counts from the ventral spinal cord, immediately caudal to the lesion, are presented in Fig. 4G, H. In the ipsilateral spinal cord, an impact of O_2_ therapy at 3 ATA was observed for small (Fig. 4G,  *p* < 0.002) as well as large neurons (Fig. 4H,  * p* < 0.002). In the rostral ipsilateral spinal cord (dorsal and ventral regions), and the caudal dorsal cord, there was a robust impact of SCI on neuronal counts, but no discernable impact of O_2_ treatment (Additional file 1: Fig. S6). In the spinal cord contralateral to the contusion impact, there was no effect of SCI or O_2_ treatment on counts of NeuN positive cells (Additional file 1: Fig. S6).

### Breathing patterns

**Fig. 5:**
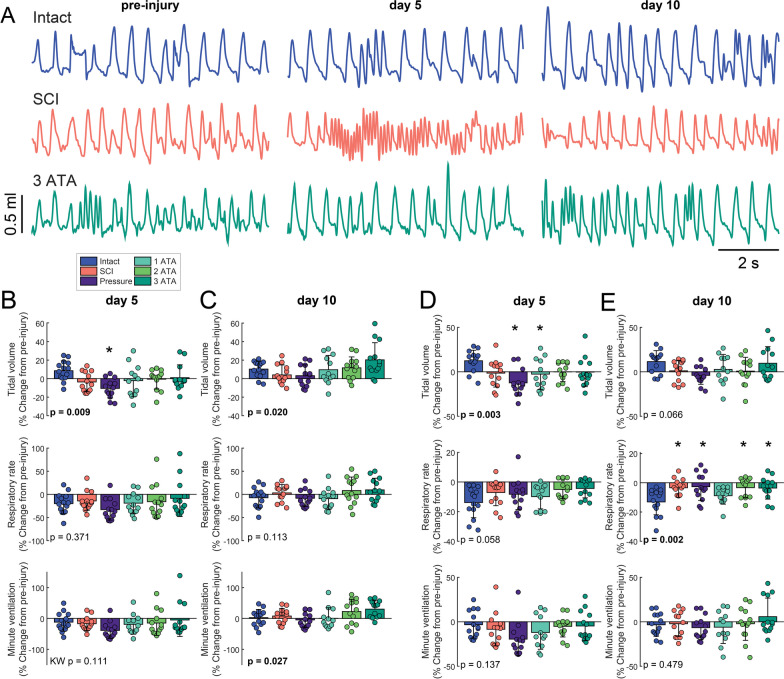
Impact of SCI and O_2_ treatment on tidal volume, rate, and minute ventilation. Examples of respiratory waveforms recorded using whole body plethysmography are shown in panel **A**. Inspiration is shown as an upward deflection. Panels **B**, **C** show mean results during normoxic (21% O_2_) breathing on day 5 and day 10 post-SCI, respectively. After normoxic baseline recordings, a brief (5-min) challenge was induced by flowing a reduced O_2_ (10%) and increased CO_2_ (7%) gas mixture through the plethysmography chamber. This was done to test the capacity to increase breathing; mean results are shown in panels **D**, **E**. A one-way analysis of variance was used to determine group differences, if a data-set failed ANOVA assumptions a Kruskal–Wallis (KW) test was run. The omnibus effect of group *p*-value is noted in the figure; *indicates *p* < 0.05 Dunnet’s post-hoc compared to Intact

Breathing was assessed using whole body plethysmography (e.g., Figs. 1D and 5A) before, 5- and 10-days after SCI. The impact of SCI on respiratory rate, tidal volume and minute ventilation during periods of “quiet” or eupneic breathing is shown in Fig. 5B, C. On the 5th post-injury day, tidal volume and respiratory rate were similar between the untreated SCI group and all of the O_2_ treatment groups (Fig. 5B). The “pressure control” group, however, displayed a reduced tidal volume. In all groups, the minute ventilation values cluster below the pre-injury values at 5-days, suggesting hypoventilation. At 10 days post-injury, minute ventilation returned to, or increased above, pre-injury values, in all SCI groups. The 3-ATA O_2_ therapy group tended to have the largest tidal volume and minute ventilation (Fig. 5C).

To assess the ability to increase breathing above baseline (“eupneic”) values, rats were given an acute respiratory challenge (5-min; 10% O_2_ and 7% CO_2_, balance N_2_). At the 5-day time point, the pressure control group showed an impaired ability to increase ventilation during the challenge, owing to a reduced tidal volume response. Ventilatory responses in the other SCI rats were similar across the experimental groups at 5-days (Fig. 5D). After 10 days, however, the 3 ATA O_2_ therapy group showed evidence of increased ability to increase tidal volume during the respiratory challenge, as compared to all other SCI groups (Fig. 5E, treatment effect, *p* = 0.066).

**Fig. 6:**
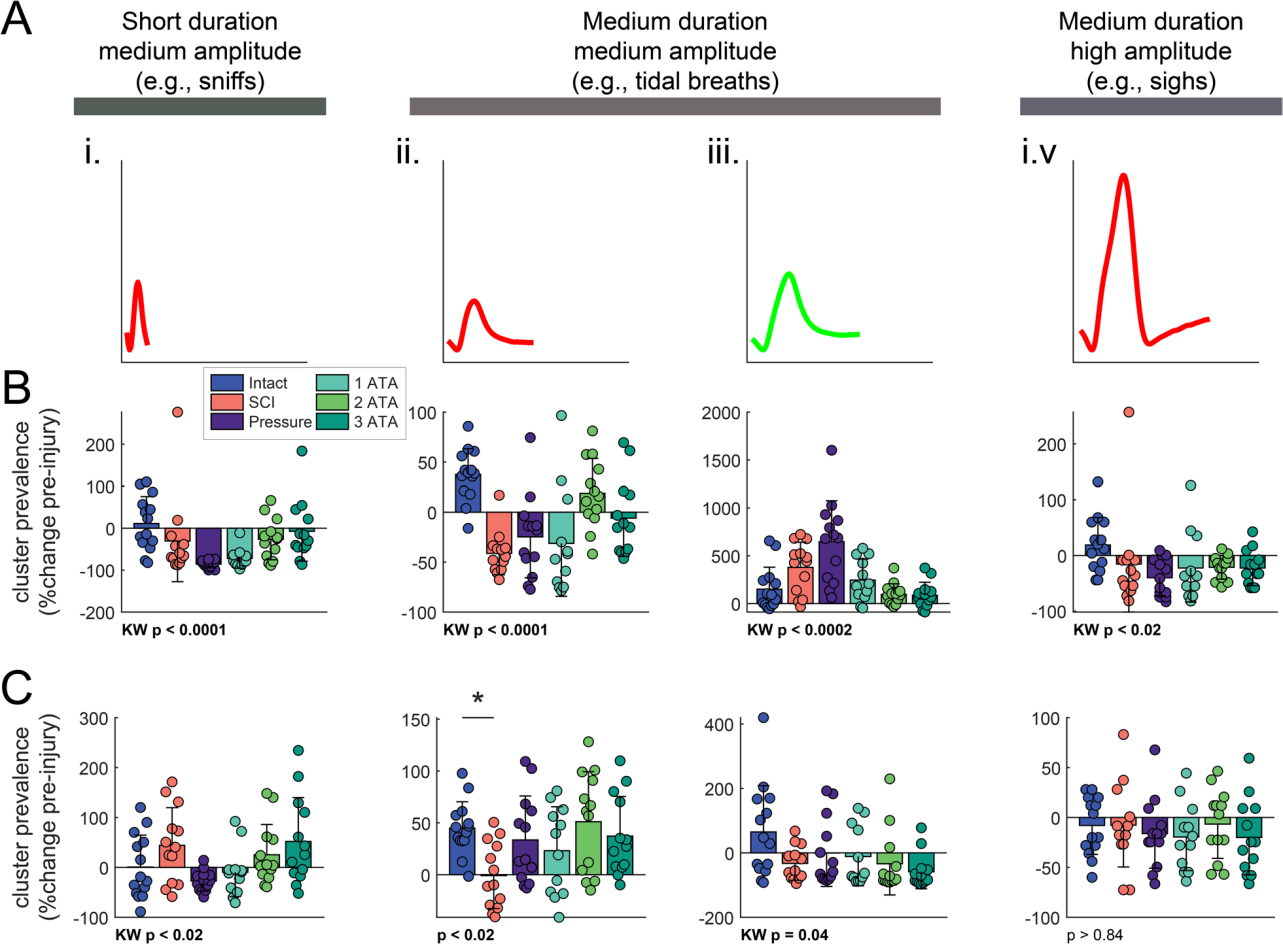
Impact of SCI and O_2_ treatment on respiratory waveforms. Cluster analysis (see methods) revealed that four general waveform categories were present during baseline (normoxic) breathing. Average waveforms from each category are shown in panel **A**. Waveform of clusters that had reduced prevalence on day 5 post-SCI are shown and red; increased prevalence is indicated by green. Mean data from 5- and 10-days post-SCI are shown in panels **B** and **C**, respectively. All data are expressed relative to the pre-injury measurement. One-way analysis of variance was used to determine group differences, if a data-set failed ANOVA assumptions a Kruskal–Wallis (KW) test was applied. The omnibus effect of group *p*-value is noted in the figure; *indicates *p* < 0.05 Dunnet’s post-hoc compared to Intact

The respiratory waveforms recorded during plethysmography (e.g., Fig. 5A) were also analyzed based on temporal appearance [18]. This analysis identified four general waveform categories as depicted in Fig. 6A. Short duration waveforms, likely indicative of “sniffing” (Fig. 6Ai), occurred less frequently at 5-days post-SCI. The distribution of “tidal breaths” also dramatically shifted after SCI. Smaller amplitude breaths (Fig. 6Aii) occurred less frequently, whereas larger amplitude breaths (Fig. 6Aiii) occurred more often. Large amplitude respiratory efforts (i.e., “sighs”, Fig. 6Aiv) occurred less frequently. Mean 5-day data are shown in Fig. 6B, with data expressed relative to the pre-SCI measurement. Note also that the laminectomy surgery (spinal intact group) also had an impact on the respiratory waveforms, with increased reliance of tidal breaths of lesser amplitude, and this differed sharply from the response to SCI. At 5-days, the prevalence of both of both the small and large tidal breaths was impacted by O_2_ therapy (Fig. 6B). As breathing ability recovered over the period of 5–10 days post-SCI, the prevalence of the various respiratory waveform also changed. At 10-days, the occurrence of small amplitude tidal breaths was elevated in all but the SCI group, and the occurrence of large amplitude tidal breaths was similar to pre-injury levels in all the SCI groups (Fig. 6C).

### Diaphragm fiber size and contractility

**Fig. 7:**
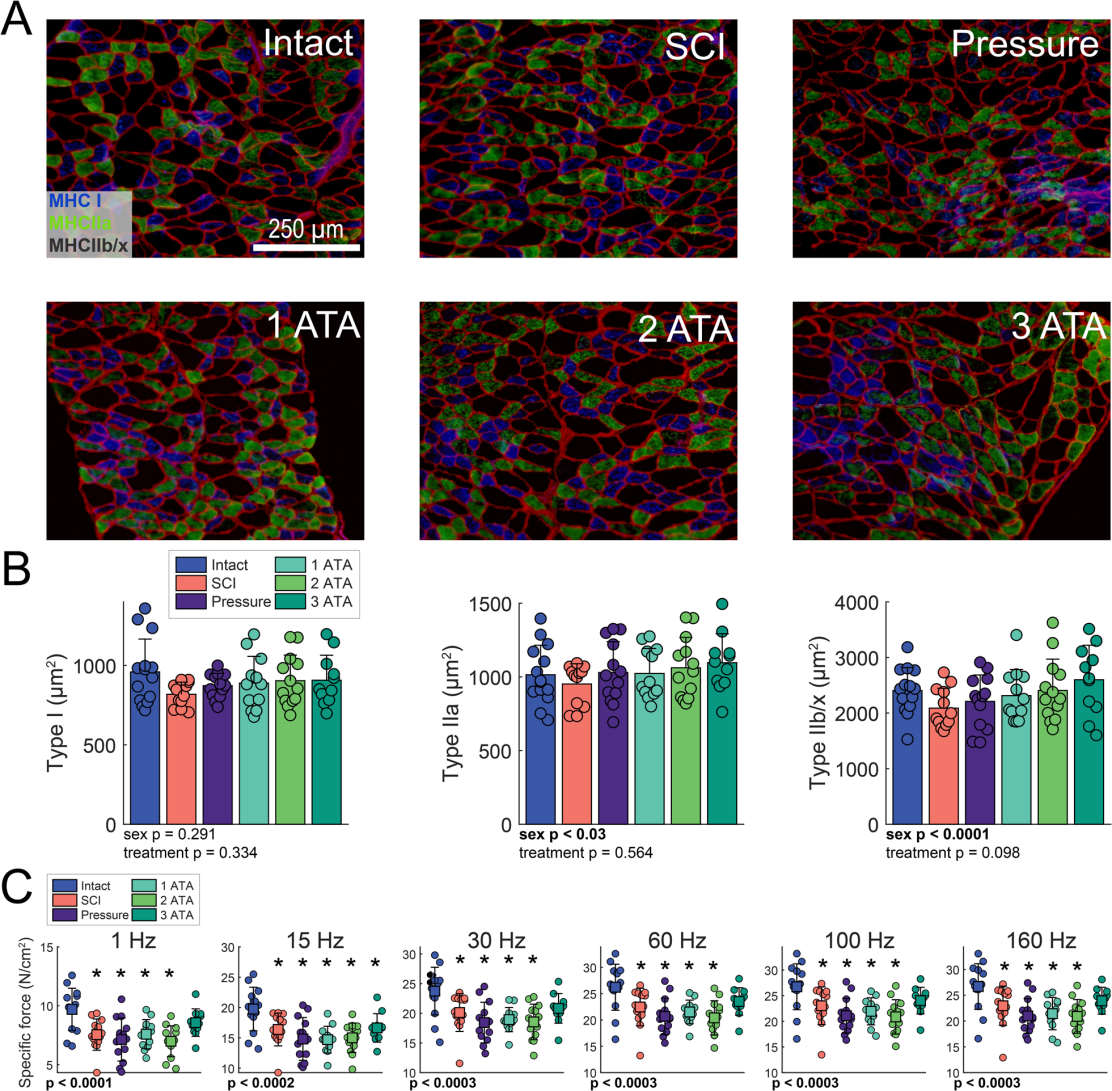
Impact of SCI and O_2_ therapy on diaphragm cross-sectional area and contractile function. **A** Representative photomicrographs of tissue sections from the mid-costal diaphragm in each experimental group. Tissues were stained to recognize myosin heavy chain I (MHC I, blue), myosin heavy chain IIa (MHC IIa, green), and laminin (red). Unstained fibers are type IIb/x. **B** Summary data of the cross sectional area. **C** Ex-vivo assessment of diaphragm specific force. A one-way ANOVA was used to determine group differences. The omnibus effect of group *p*-value is noted; *indicates *p* < 0.05 Dunnet’s post-hoc compared to Intact

The diaphragm was immunohistologically evaluated to determine myofiber type and size; representative photomicrographs are shown in Fig. 7A. As myofiber size is sex dependent we evaluated the cross-sectional area with a two-way ANOVA (sex and treatment Fig. 7B). Indeed, there was an effect of sex (*p* < 0.03) in both the type IIa and type IIb/x fibers. Diaphragm myofiber size in spinal intact rats showed considerable variability as shown in Fig. 7B. After SCI, type I fiber size clustered more tightly in the untreated (SCI) and pressure control groups, with values below the mean of the spinal intact condition. There was not a consistent impact of O_2_ therapy on type I fiber size, and all O_2_ treated rats showed variability similar to the spinal intact condition (Fig. 7B). Type IIa fiber size was similar across all experimental groups (Fig. 7B). Type IIb/x fiber size clustered below the spinal intact mean in the SCI control (no treatment) group. A trend was observed for an increase in size of the IIb/x myofibers in the 3 ATA O_2_ therapy groups (two-way ANOVA, treatment *p* = 0.098). Thus, the null hypothesis (i.e., no difference in type IIb/x fiber size across groups) is not accepted with any confidence.

Muscle contractility was assessed ex vivo using strips from the costal diaphragm. Diaphragm specific force was reduced following SCI, an effect that was present across all stimulation frequencies (Fig. 7C). There was indication of a modest impact of 3 ATA O_2_ treatment at 1 Hz, and 30–160 Hz stimulation frequencies. In all cases, one-way ANOVA indicated a robust effect of treatment (*p* < 0.0001 at each frequency tested), and post-hoc comparisons detected no difference between 3 ATA O_2_ therapy group and the spinal intact condition at the aforementioned stimulation frequencies.

## Discussion

This work demonstrates that HBO, but also non-pressurized O_2_ therapy, when delivered over the initial days following cervical SCI, has a substantial impact on the injured spinal cord. Consistent with prior reports, HBO therapy initiated early after SCI had an anti-inflammatory impact on the injured spinal cord. However, pressurizing the hyperoxic gas does not appear necessary for the anti-inflammatory effect of O_2_ therapy since a similar response occurred when O_2_ was delivered at normobaric pressure.. The 3 ATA group also showed evidence of increased inspiratory tidal volume as compared to the other O_2_ therapy groups. When taking all outcome measures into consideration, HBO therapy delivered 3 ATA pressure had the most benefit, but 100% O_2_ exposure at 1 ATA also had a beneficial impact, and thus merits further study as a therapeutic acutely post-SCI.

### HBO therapy after SCI

In 1972, Kelly et al. demonstrated that SCI in adult dogs resulted in a rapid drop of O_2_ levels in the immediate vicinity of the spinal lesion [1]. An intraspinal O_2_ sensing electrode was used to demonstrate that local tissue partial pressure of O_2_ (PO_2_) dropped to almost zero immediately after SCI. Exposure to HBO rapidly increased spinal PO_2_ to well above pre-injury levels, and importantly, this effect did not happen with normobaric 100% O_2_ treatment. Dogs in the HBO treatment group also had improved neurologic recovery after the SCI [1]. A few years after that report, studies in sheep showed that HBO therapy delivered acutely after SCI could mitigate spinal histopathology and improve motor recovery [36, 37]. Recent studies of HBO therapy in rodent SCI models have demonstrated reductions in oxidative stress [38, 39] and apoptosis [40] in the injured spinal cord, preserved skeletal muscle function [7] and improved motor recovery [41]. Proteomic analysis of the injured rat spinal cord after HBO treatment has revealed increased expression of proteins associated with O_2_ transport and binding [42].

There have been multiple clinical trials of HBO therapy in spinally injured humans, with treatments initiated as early as 24 h post-injury [8–13, 43]. There is also an ongoing clinical trial in Australia (https://clinicaltrials.gov/show/NCT03101982). Neurological benefit has been reported in the majority of published HBO clinical trials. However, skepticism remains [44], and in our opinion this is at least in part due to tremendous variability in regards to the patient population, outcome measures, and HBO paradigms (dose, duration, timing post-injury) in human clinical studies. In this regard, a recent review of HBO clinical trials in SCI to date commented that the quality of the trials has been “poor” and “further studies are needed” [45]. Currently HBO continues to be used in clinical treatment, as evidenced by a recent case study of an American football player who suffered a SCI during competition [46]. The patient showed remarkable recovery, although it was not possible to attribute the recovery to HBO therapy vs. other aspects of his clinical care.

### Mechanisms of O_2_ therapy after SCI

One very likely impact of O_2_ therapy is mitigation of local tissue hypoxia after SCI [1, 2]. Raising inspired O_2_ from “room air” levels (21%) to 100% will drive the arterial partial pressure of O_2_ (PaO_2_) from 100 to > 500 mmHg. In turn, this will increase plasma O_2_ content by approximately fourfold, from 0.5 to 2.3 mL O_2_ per 100 ml plasma. Pressuring 100% O_2_ to 3 ATA will further raise PaO_2_ > 2000 mmHg, and plasma O_2_ will reach > 14-fold of “normal” to contain approximately 7 ml O_2_ per 100 mL. This level of plasma O_2_ is sufficient to support metabolic need without contribution from hemoglobin bound O_2_. Thus, O_2_ diffusion gradients (i.e., capillary plasma to interstitial fluid) will increase dramatically with O_2_ therapy, and HBO will create a substantially larger gradient as compared to normobaric O_2_ therapy.

Increasing O_2_ delivery after SCI is important because tissue PO_2_ near a spinal lesion acutely drops close to zero [1], and remains very low even months after injury [2]. Acutely, the low spinal PO_2_ is secondary to the physical interruption of vasculature resulting from the spinal trauma. While spinal cord revascularization occurs to an extent after chronic SCI, there remains a persistent vascular dysregulation due to pericyte-induced constriction of capillaries [2]. This pericyte mechanism, and possibly reduced arterial blood pressure [47], are likely contributors to persistent spinal cord hypoxia in the chronically injured spinal cord. Increasing the gradient for diffusion of O_2_ from the vasculature to areas surrounding a spinal lesion—thereby increasing local O_2_ availability—may be the reason underlying the impact of HBO therapy on neuronal survival / necrosis noted here (e.g., Fig. 4) and in prior studies [6, 36, 37, 40].

An anti-inflammatory effect of HBO therapy has been demonstrated after SCI [4, 6] and also after brain injury [48, 49]. Further, HBO treatment can suppress NF-κB signaling after SCI [4, 6]. This is important, since NF-κB expression drives pro-inflammatory signaling pathways via activation of cytokines and chemokines [50]. Here we confirm an anti-inflammatory impact of HBO therapy after SCI as reflected by the RNAseq gene module assessment as well as IBA-1 and GFAP spinal immunochemistry. Further, plasma levels of an anti-inflammatory cytokine (IL-4) [51] were upregulated after 3 ATA HBO treatment. IL-4 can improve recovery following brain injury [52] or SCI [53]. When rats with thoracic (T8) contusion injury were treated with IL-4, it produced an increase in serum anti-inflammatory cytokine levels, increased neuronal counts near the lesion, and improved functional recovery [53].

Our results using normobaric 100% O_2_ (included in our study as a control group) are particularly informative regarding the inflammatory response. Two different outcome measures (RNAseq gene module assessment and spinal immunochemistry) indicated a powerful impact of daily normobaric O_2_ treatment. Accordingly, the anti-inflammatory impact of HBO may not require increased atmospheric pressure during hyperoxia. Rather, normobaric hyperoxia treatments are sufficient to trigger at least some of the anti-inflammatory mechanisms associated with HBO. An increase in plasma IL-4, however, was unique to 3 ATA HBO treatment and did not occur with normobaric hyperoxia. On the other hand, plasma IL-17a levels increased after normobaric hyperoxia. This may have benefit, as there is evidence that IL-17a promotes neural precursor cell survival, synapse formation, and recovery after stroke [54, 55]. Conversely, acutely post stroke, IL-17a contributes to the inflammatory cascade [56]. The current data were obtained at 11-day post SCI, which is likely to be outside the acute window of IL-17a mediated inflammatory responses.

### Respiratory function and recovery after cervical SCI

Approximately half of all injuries to the spinal cord occur in the cervical region [57]. Cervical SCI always produces some degree of respiratory-related complications, the severity of which correlates with the segmental level of injury [58]. Further, respiratory dysfunction is a frequent cause of re-hospitalization [59] and the leading cause of mortality after SCI [60]. Thus, any treatment which can improve breathing ability has the potential for improving quality of life in persons with SCI. For this reason, we focused on breathing-related outcomes in our study of O_2_ therapy after cervical SCI.

In the current study there was a strong recovery of breathing by 10-days following cervical SCI, even in rats that did not receive O_2_ therapy. Prior studies using cervical contusion injury, as used here, report that during quiet breathing a transient deficit in diaphragm muscle activation occurs. This deficit, however, is followed by spontaneous recovery over a period of days to weeks [61–64]. Thus, the recovery of tidal volume and ventilation observed during baseline (“eupnea”) conditions was expected. This spontaneous recovery presumably occurs due to activation of spared bulbospinal pathways to spinal respiratory motoneurons as well plasticity in propriospinal networks [14]. In rodent cervical SCI models, deficits in diaphragm activation and tidal volume generation are typically most evident during conditions that require increased ventilation. For this reason, we evaluated breathing during an acute hypoxic-hypercapnic “respiratory challenge”. During the challenge, of the experimental groups with SCI, the 3 ATA HBO group had the largest inspiratory tidal volume. This result suggests an increased ability to activate the respiratory muscles. This could occur due to a greater number of surviving neurons (e.g., Fig. 4) or improved diaphragm contractility (e.g., Fig. 7).

### Diaphragm function after HBO

Prior work demonstrates an impact of HBO treatment on skeletal muscle, including improved regeneration after injury (for review, see [65]). In regards to cervical SCI, a prior study showed that HBO treatment (3 ATA, 1-h per day, 10-consecutive days) increased the specific force production of the diaphragm and attenuated myofiber atrophy [7]. Further, these responses were associated with increases in diaphragm antioxidants as well as a reduction atrophy-related gene expression. The current study produced similar results in that the 3 ATA HBO group had evidence of preserved type IIb/x myofiber size and increased contractility. These changes, however, were not noted in the 1 ATA O_2_ therapy group. Thus, after SCI, the benefits of hyperoxia therapy on the diaphragm appear to require elevations in ambient pressure.

### Two vs. three ATA HBO

The impact of 2 ATA HBO treatment in our study is difficult to explain. Across several outcome measures assessing inflammation (e.g., RNAseq, spinal immunohistochemistry), the 1 and 3 ATA O_2_ therapy groups appeared to perform better than the group exposed to hyperoxia at 2 ATA. Further, there was increased variability across several outcome measures (e.g., RNAseq, spinal immunohistochemistry, myofiber CSA type IIb/x, and specific force. This effect was also reproducible across cohorts as the RNAseq experiments were run separately from all other cohorts. One possibility is that increasing ambient pressure can impair recovery, but the 3 ATA treatment overcomes any pressure evoked “negative mechanisms” by flooding the lesion area with high levels of O_2_. This is speculation, however, and this observation merits further study.

### Conclusions

HBO therapy for SCI has been described in the literature for > 50 years [1], and is still used in persons with SCI [46]. However, HBO is has not been widely adopted as a therapeutic approach in SCI, as a deeper understanding of the underlying physiological mechanisms and functional impact is needed. In this regard, our study contributes several novel findings. With regard to neuroinflammation, our data confirm prior reports of the anti-inflammatory impact of HBO. But more importantly, we conclude that normobaric O_2_ therapy after SCI can also reduce the spinal inflammatory response. This suggests that the increased levels of plasma O_2_ that will occur with HBO is not needed for the anti-inflammatory benefit. With regard to neuroprotection, however, we conclude that pressuring O_2_ to 3 ATA was necessary to minimize neuronal loss in our lesion model. This conclusion applies only to the 1-h treatment paradigms acutely after injury as studied here. It is certainly possible that normobaric O_2_ therapy at longer durations could also evoke neuroprotection. Overall, we conclude that with daily 1-h exposures following cervical SCI, normobaric O_2_ treatment can reduce the spinal inflammatory response, but pressured O_2_ (i.e., HBO) provides further benefits.

### Supplementary Information

**Additional file 1: Figure S1.** Heatmaps depicting RNAseq results. Data were generated by performing RNAseq on C4 spinal tissues, followed by hierarchical clustering of Weighted Gene Co-Expression Network Analysis (WGCNA) module eigengenes. Depicted is the expression of top 25 hub genes (rows) across individual animals (columns) for each of the six experimental treatment groups. Red corresponds to gene upregulation and blue to downregulation. Bar plots above the heat maps show the overall expression level within each animal. See Figure 1 for a summary of the functional classification of all identified modules, as well as the heatmaps for the two modules most strongly impacted by oxygen therapy. **Figure S2.** Luminex immunoassay of circulating cytokines/chemokines. Plasma levels of eotaxin, interleukin-1beta (IL-1b), interleukin-18 (IL-18), fractalkine, granulocyte-macrophage colony-stimulating factor (GM-CSF), interleukin-10 (IL-10), monocyte chemoattractant protein-1 (MCP-1), C-X-C motif chemokine 5 (LIX), leptin, interleukin-5 (IL-5), interferon gamma-induced protein 10 (IP-10), tumor necrosis factor-alpha (TNFa), interleukin-4 (IL-4), interleukin-17a (IL-17a), vascular endothelial growth factor (VEGF), regulated upon activation, normal T cell expressed and presumably secreted (RANTES). A one-way analysis of variance was used to determine group differences, if a data-set failed ANOVA assumptions a Kruskal–Wallis (KW) test was run. The omnibus effect of group p-value is noted in the figure ; * indicates p<0.05 Tukey-Kramer post-hoc compared to Intact. **Figure S3.** Examples of cresyl violet stained spinal cords. One example from each experimental group is included. **Figure S4.** Oxygen therapy reduces neuroinflammation. Assessment of iba1+ optical density across the entire rostral caudal axis on the A) ipsilateral and B) contralateral sides of the spinal cord. In the SCI group there is elevated iba1^+^ staining in the SCI group that extends throughout the whole area (-10 to +10 mm) examined. Assessment of GFAP^+^ optical density across the rostral caudal axis (-10 to +10 mm from epicenter) on the C) ipsilateral and D) contralateral sides of the spinal cord. E) Example photomicrographs near the lesion epicenter for all experimental groups. **Figure S5.** Oxygen therapy mitigates vacuolization. Rostral caudal assessment of vacuolization. To determine the impact of injury and treatment, we assessed tissue damage on the A) ipsilateral and B) contralateral sides of the spinal cord. Further we quantified tissue sparing 3 mm rostral and 3 mm caudal to the epicenter as indicated by the gray box. C) Example photomicrographs near the lesion epicenter for all experimental groups. **Figure S6.** Neuronal counts. A) In the dorsal caudal cord ipsilateral to contusion there was no discernable effect of injury or treatment. B) rostral to the injury epicenter (-2000 to -500 µm) injury led to a reduction in both small (23-115 µm^2^) and large neurons (116-345 µm^2^) with no effect of oxygen therapy. C) Contralateral to injury (-2000 to 2000 µm) there was no impact of injury or treatment. A one-way analysis of variance was used to determine group differences. The omnibus effect of group p-value is noted in the figure; * indicates p<0.05 Dunnet’s post-hoc compared to Intact. D) Example photomicrographs near the lesion epicenter for all experimental groups.

## Data Availability

Matlab code used in the analyses, and the RNAseq datasets are available at https://odc-sci.org/. All other data are available upon reasonable request to the corresponding author.
