# Supplementary material for: Oxygen therapy attenuates neuroinflammation after spinal cord injury
Source: J Neuroinflammation. 2023 Dec 19;20:303. doi: 10.1186/s12974-023-02985-6 (PMC10729514; doi:10.1186/s12974-023-02985-6)
Supplement: Supplementary file 1 — Additional file 1: Figure S1. Heatmaps depicting RNAseq results. Data were generated by performing RNAseq on C4 spinal tissues, followed by hierarchical clustering of Weighted Gene Co-Expression Network Analysis (WGCNA) module eigengenes. Depicted is the expression of top 25 hub genes (rows) across individual animals (columns) for each of the six experimental treatment groups. Red corresponds to gene upregulation and blue to downregulation. Bar plots above the heat maps show the overall expression level within each animal. See Figure 1 for a summary of the functional classification of all identified modules, as well as the heatmaps for the two modules most strongly impacted by oxygen therapy. Figure S2. Luminex immunoassay of circulating cytokines/chemokines. Plasma levels of eotaxin, interleukin-1beta (IL-1b), interleukin-18 (IL-18), fractalkine, granulocyte-macrophage colony-stimulating factor (GM-CSF), interleukin-10 (IL-10), monocyte chemoattractant protein-1 (MCP-1), C-X-C motif chemokine 5 (LIX), leptin, interleukin-5 (IL-5), interferon gamma-induced protein 10 (IP-10), tumor necrosis factor-alpha (TNFa), interleukin-4 (IL-4), interleukin-17a (IL-17a), vascular endothelial growth factor (VEGF), regulated upon activation, normal T cell expressed and presumably secreted (RANTES). A one-way analysis of variance was used to determine group differences, if a data-set failed ANOVA assumptions a Kruskal–Wallis (KW) test was run. The omnibus effect of group p-value is noted in the figure ; * indicates p<0.05 Tukey-Kramer post-hoc compared to Intact. Figure S3. Examples of cresyl violet stained spinal cords. One example from each experimental group is included. Figure S4. Oxygen therapy reduces neuroinflammation. Assessment of iba1+ optical density across the entire rostral caudal axis on the A) ipsilateral and B) contralateral sides of the spinal cord. In the SCI group there is elevated iba1+ staining in the SCI group that extends throughout the whole area (-10 to +10 [file 12974_2023_2985_MOESM1_ESM.docx]

**ONLINE DATA**

**Oxygen therapy attenuates neuroinflammation after spinal cord injury**

Michael D. Sunshine^1,2^, Victoria E. Bindi^1,2^, Branden L Nguyen^5^, Vivian Doerr^5^, Franccesco P. Boeno^5^, Vijayendran Chandran^4^, Ashley J. Smuder^2,5^, *David D. Fuller^1,2,3^

^1^Dept. of Physical Therapy, University of Florida

^2^Breathing Research and Therapeutics Center, University of Florida

^3^McKnight Brain Institute, University of Florida

^4^Dept. of Pediatrics, University of Florida

^5^Dept. of Applied Physiology and Kinesiology, University of Florida

***Correspondence**:

David D. Fuller

Email: dfuller@phhp.ufl.edu

Telephone: 352 273 6634

Fax: 352 273 6109

**FIGURES**

| 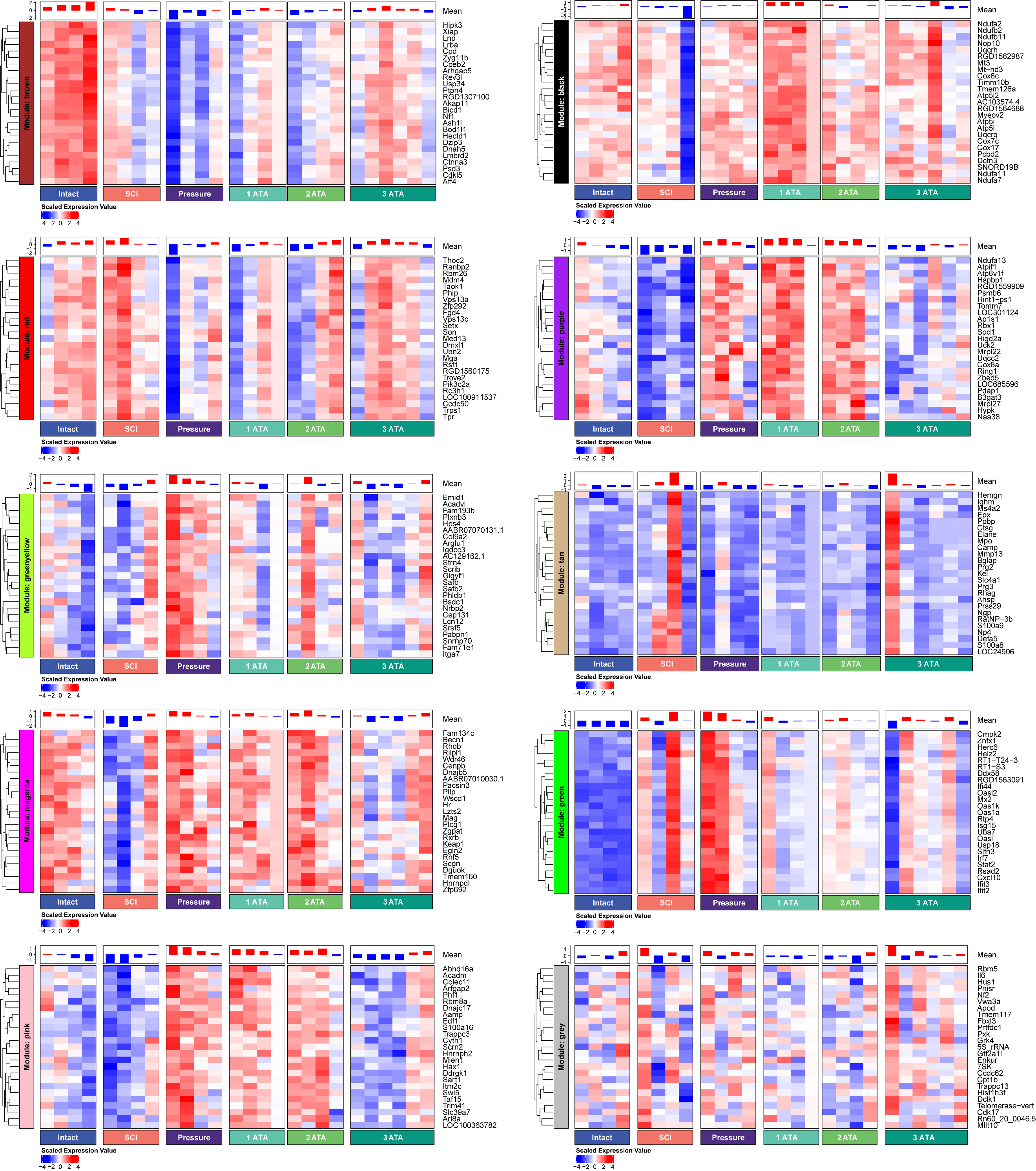  **Figure S1. Heatmaps depicting RNAseq results.** Data were generated by performing RNAseq on C4 spinal tissues, followed by hierarchical clustering of Weighted Gene Co-Expression Network Analysis (WGCNA) module eigengenes. Depicted is the expression of top 25 hub genes (rows) across individual animals (columns) for each of the six experimental treatment groups. Red corresponds to gene upregulation and blue to downregulation. Bar plots above the heat maps show the overall expression level within each animal. See Figure 1 for a summary of the functional classification of all identified modules, as well as the heatmaps for the two modules most strongly impacted by oxygen therapy. |
| --- |

| 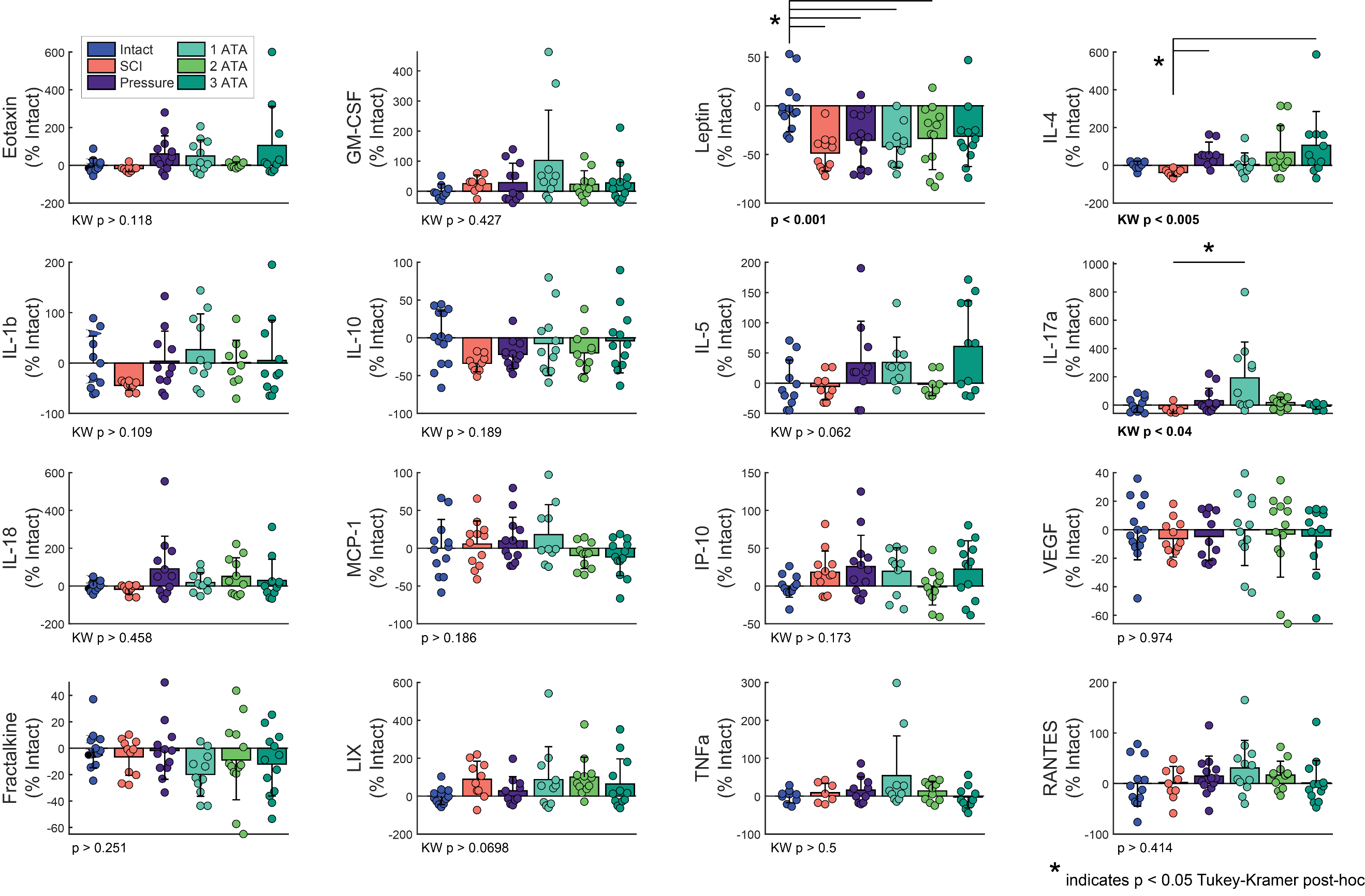  **Figure 2. Luminex immunoassay of circulating cytokines/chemokines.** Plasma levels of eotaxin, interleukin-1beta (IL-1b), interleukin-18 (IL-18), fractalkine, granulocyte-macrophage colony-stimulating factor (GM-CSF), interleukin-10 (IL-10), monocyte chemoattractant protein-1 (MCP-1), C-X-C motif chemokine 5 (LIX), leptin, interleukin-5 (IL-5), interferon gamma-induced protein 10 (IP-10), tumor necrosis factor-alpha (TNFa), interleukin-4 (IL-4), interleukin-17a (IL-17a), vascular endothelial growth factor (VEGF), regulated upon activation, normal T cell expressed and presumably secreted (RANTES). A one-way analysis of variance was used to determine group differences, if a data-set failed ANOVA assumptions a Kruskal–Wallis (KW) test was run. The omnibus effect of group p-value is noted in the figure ; * indicates p<0.05 Tukey-Kramer post-hoc compared to Intact. |
| --- |


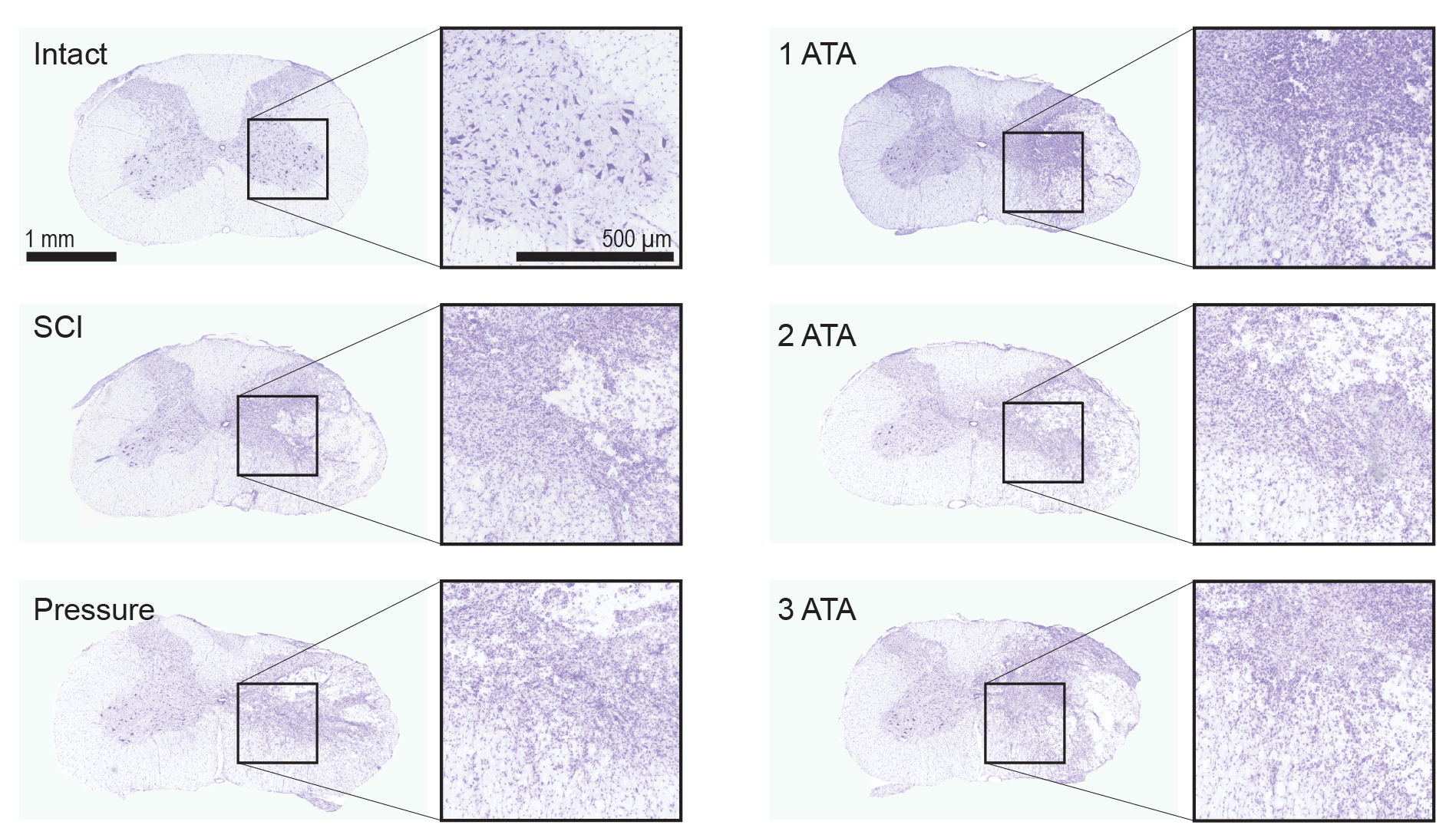


**Figure S3. Examples of cresyl violet stained spinal cords.** One example from each experimental group is included.

| 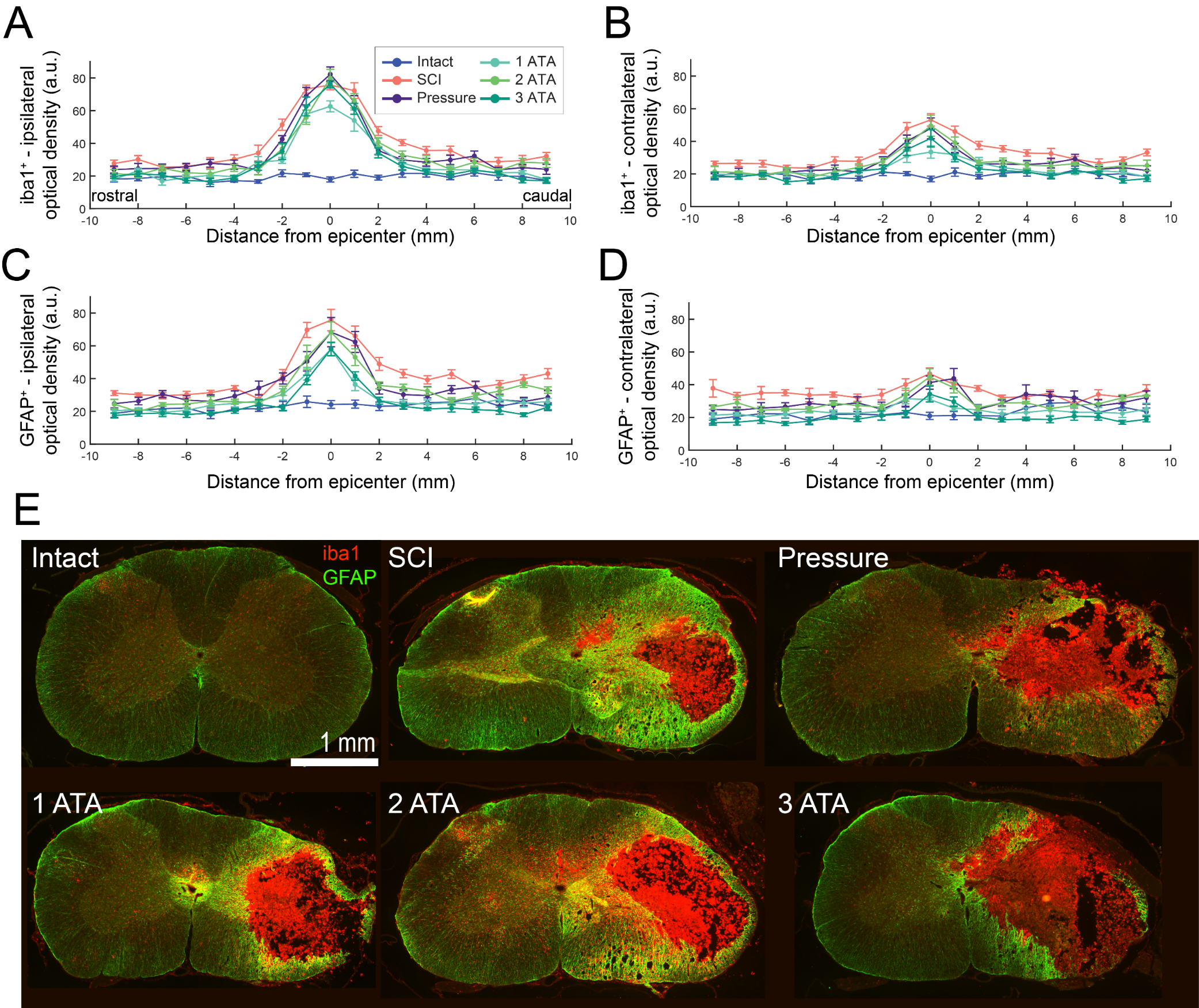  **Figure S4. Oxygen therapy reduces neuroinflammation.** Assessment of iba1+ optical density across the entire rostral caudal axis on the **A)** ipsilateral and **B)** contralateral sides of the spinal cord. In the SCI group there is elevated iba1^+^ staining in the SCI group that extends throughout the whole area (-10 to +10 mm) examined. Assessment of GFAP^+^ optical density across the rostral caudal axis (-10 to +10 mm from epicenter) on the **C)** ipsilateral and **D)** contralateral sides of the spinal cord. **E)** Example photomicrographs near the lesion epicenter for all experimental groups. |
| --- |

| 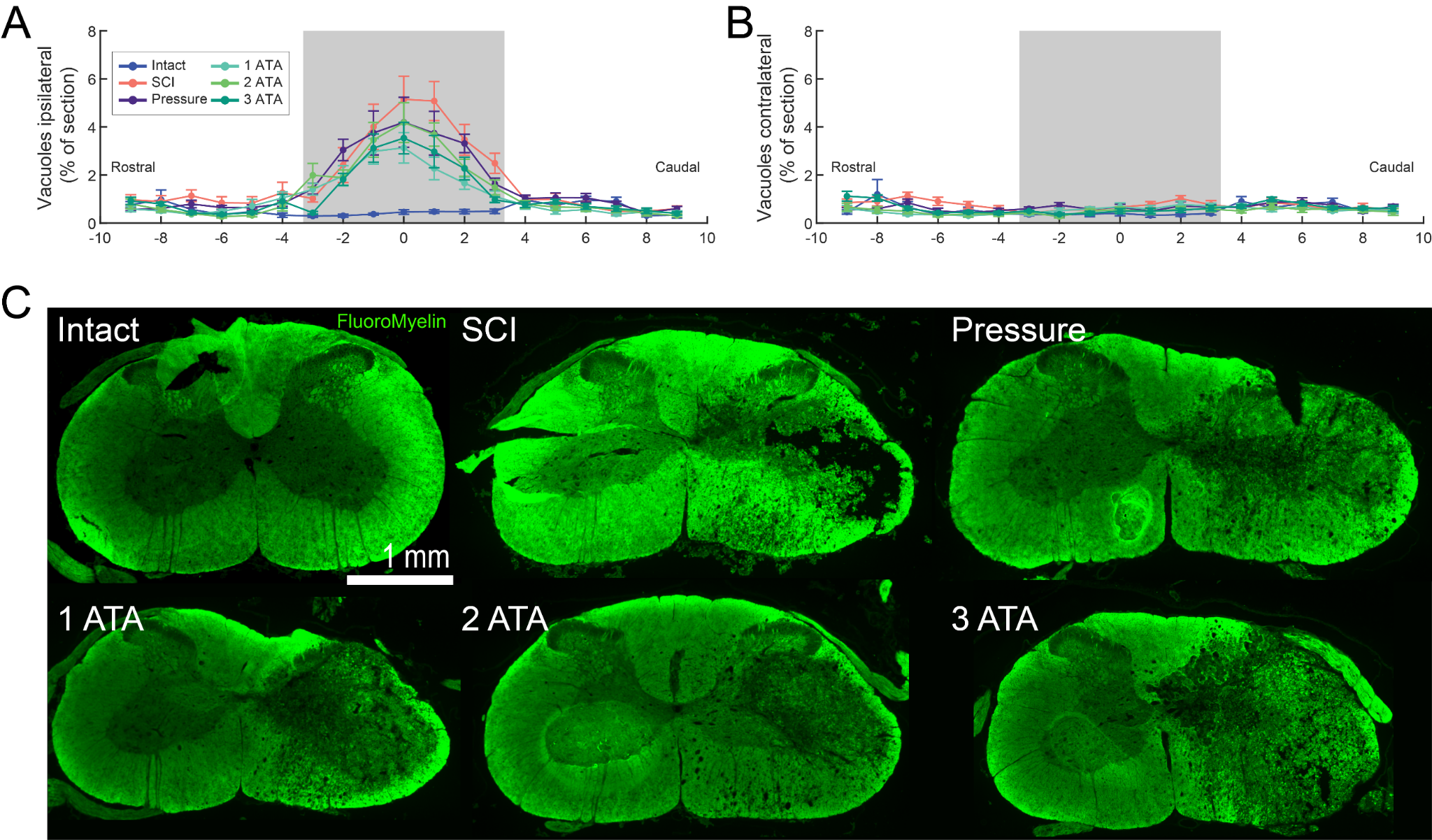  **Figure S5. Oxygen therapy mitigates vacuolization.** Rostral caudal assessment of vacuolization. To determine the impact of injury and treatment, we assessed tissue damage on the **A)** ipsilateral and **B)** contralateral sides of the spinal cord. Further we quantified tissue sparing 3 mm rostral and 3 mm caudal to the epicenter as indicated by the gray box. **C)** Example photomicrographs near the lesion epicenter for all experimental groups. |
| --- |

| 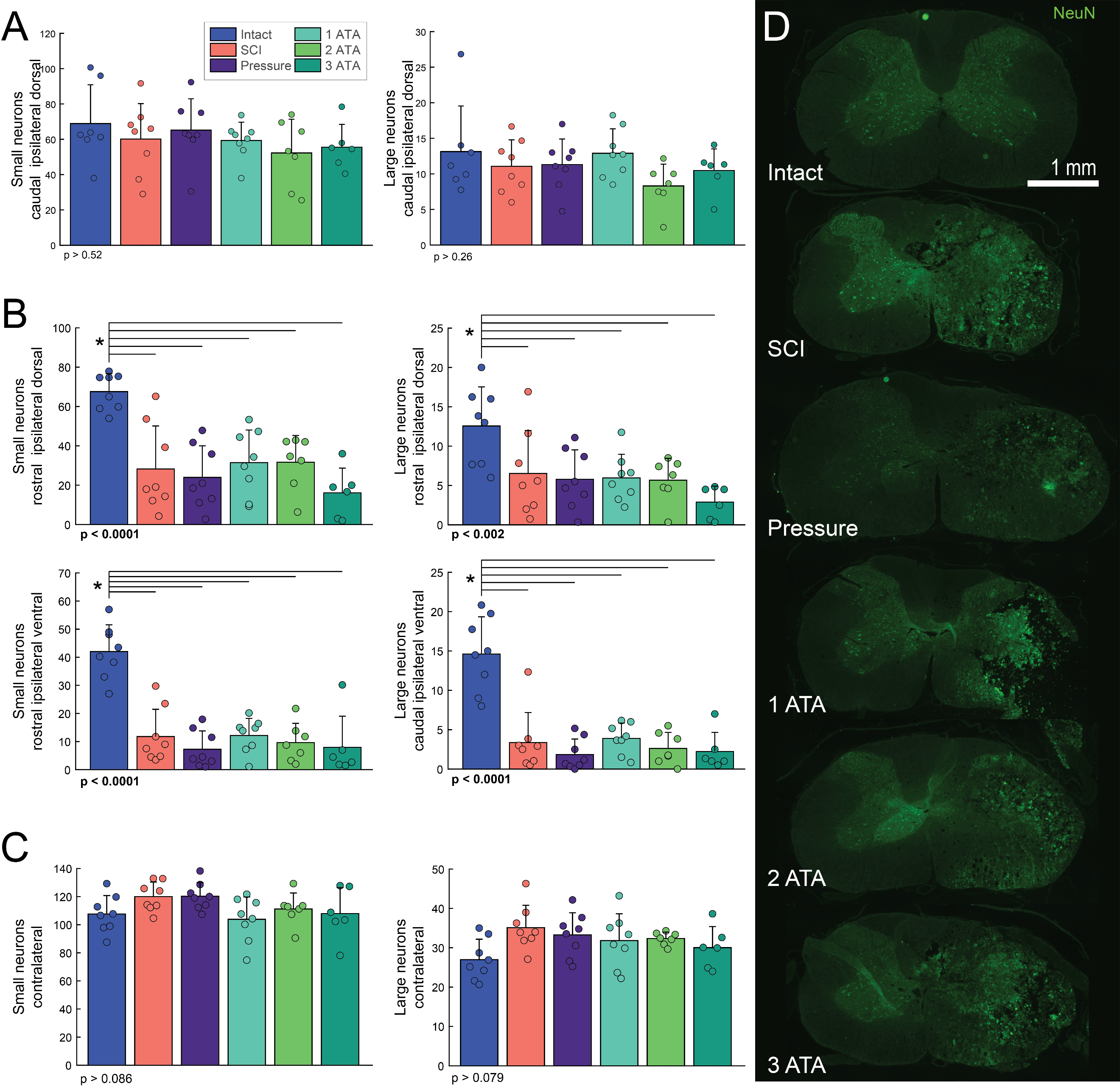  **Figure S6. Neuronal counts.** **A)** In the dorsal caudal cord ipsilateral to contusion there was no discernable effect of injury or treatment. **B)** rostral to the injury epicenter (-2000 to -500 µm) injury led to a reduction in both small (23-115 µm^2^) and large neurons (116-345 µm^2^) with no effect of oxygen therapy. **C)** Contralateral to injury (-2000 to 2000 µm) there was no impact of injury or treatment. A one-way analysis of variance was used to determine group differences. The omnibus effect of group p-value is noted in the figure; * indicates p<0.05 Dunnet’s post-hoc compared to Intact. **D)** Example photomicrographs near the lesion epicenter for all experimental groups. |
| --- |
